# Cadmium-Induced antioxidant gene expression in *Corbicula fluminea*: a cross-sectional study in the Euphrates River, Iraq

**DOI:** 10.11604/pamj.2026.53.117.49816

**Published:** 2026-03-09

**Authors:** Kadhum Sabah Nasir, Nacim Louhichi

**Affiliations:** 1 Department of Sciences of Sfax, Faculty of Sciences of Sfax, University of Sfax, Soukra Road km 4 - BP 1171-3000 Sfax, Tunisia,; 2 Department of Medicine of Sfax, Faculty of Medicine of Sfax, Laboratory of Molecular and Cellular Screening Processes, Center of Biotechnology of Sfax, University of Sfax, Sidi Mansour Road Km 6, P.O. Box 1177, Sfax 3018, Tunisia

**Keywords:** Cadmium, *Corbicula fluminea*, antioxidant gene expression, Euphrates River, oxidative stress, public health, heavy metals

## Abstract

**Introduction:**

Cadmium (Cd) is a toxic heavy metal prevalent in aquatic ecosystems, with known adverse effects on organisms through oxidative stress. Corbicula fluminea (Asian clam), a bioindicator species, is sensitive to environmental pollutants, including cadmium. This study investigates the spatial variation in cadmium exposure and its impact on antioxidant gene expression in Corbicula fluminea (C. fluminea) from the Euphrates River, Iraq.

**Methods:**

a cross-sectional study was conducted across three distinct sites with varying anthropogenic impacts (urban, agricultural, and aquaculture) along a 26-kilometre stretch of the Euphrates River. Cadmium concentrations in water and mollusc tissues were quantified using atomic absorption spectroscopy. The expression levels of antioxidant genes (MT, GST, CAT, SOD) were assessed via quantitative PCR. Statistical analyses included one-way ANOVA and Pearson correlation.

**Results:**

cadmium concentrations differed significantly among the sampling sites (p = 0.003), with the highest levels recorded in the urban area of Nasiriya. However, no significant differences were found in the expression levels of MT, GST, CAT, and SOD genes among the sites (all p > 0.05). Similarly, no significant correlations were observed between cadmium concentrations and gene expression.

**Conclusion:**

although cadmium contamination varied across sites, antioxidant gene responses in C. fluminea were not statistically significant. These findings suggest that gene expression responses in natural environments may be modulated by multiple interacting environmental factors, emphasising the need for broader and longitudinal ecological assessments. The integration of C. fluminea into environmental monitoring programs should therefore incorporate multiple biomarkers rather than relying solely on antioxidant gene expression.

## Introduction

Cadmium (Cd) is a toxic heavy metal that presents significant environmental and public health risks due to its persistence in ecosystems [1]. Recent global assessments estimate that cadmium contamination affects more than 10% of monitored freshwater bodies worldwide, primarily due to industrial discharge and agricultural runoff, posing significant risks to aquatic biodiversity and human health. It accumulates in freshwater systems through anthropogenic activities like industrial effluents, agricultural runoff, and improper waste management [2]. Cadmium induces oxidative stress by generating reactive oxygen species (ROS), which damage cellular components such as lipids, proteins, and DNA, impairing cellular function and threatening organismal health.

*Corbicula fluminea* (Asian clam) is a widely recognised bioindicator species used in freshwater ecotoxicology due to its high filtration rate, sediment interaction, and capacity to bioaccumulate heavy metals. Recent studies (2022-2024) highlight its ecological importance in monitoring trace metal contamination and assessing pollutant bioavailability in riverine ecosystems. Its broad geographic distribution and tolerance to environmental stressors make it particularly suitable for evaluating chronic metal exposure in natural systems [3]. As a filter feeder, it accumulates waterborne pollutants like cadmium, making it an effective species for monitoring the health of aquatic ecosystems [4]. Exposure to cadmium triggers physiological responses, including changes in the expression of antioxidant genes such as metallothionein (MT), glutathione-S-transferase (GST), superoxide dismutase (SOD), and catalase (CAT). These genes play a critical role in detoxifying ROS and mitigating cadmium's toxic effects.

Although laboratory studies have demonstrated cadmium-induced alterations in antioxidant gene expression in *C. fluminea*, field-based evidence remains limited, particularly in complex natural environments such as the Euphrates River. Unlike controlled laboratory settings, natural ecosystems involve multiple interacting stressors that may modulate molecular responses. The Euphrates, a vital water source for millions, faces significant cadmium contamination due to industrial, agricultural, and urban activities [5]. However, the biological impacts of cadmium on local species remain poorly understood [6]. Understanding how *C. fluminea* responds to cadmium exposure in such a complex, multi-stressor environment is crucial for assessing its role as a reliable bioindicator for water quality. Given the complexity of natural ecosystems, field-based studies are essential to capture the nuanced molecular responses of *C. fluminea* to cadmium exposure [7]. This study focuses on the Euphrates River, where cadmium contamination is a known issue, yet its biological and ecological consequences are underexplored.

The primary objective of this study is to investigate the spatial variation in cadmium exposure along the Euphrates River and assess its impact on antioxidant gene expression in *C. fluminea*. This study aims to provide insights into how cadmium accumulates in this species and evaluate its potential as a sentinel organism for monitoring cadmium contamination in freshwater ecosystems. Specific objectives include: quantify cadmium concentrations in water and *C. fluminea* tissues across three sites with varying levels of anthropogenic impact (urbanised, agricultural, and aquaculture-influenced areas); assess the expression of antioxidant genes (MT, GST, CAT, SOD) in *C. fluminea* specimens from these sites; evaluate whether changes in gene expression correlate with environmental cadmium concentrations and investigate site-specific differences in gene expression and cadmium accumulation to explore how local environmental conditions may influence biological responses. By achieving these objectives, this study will contribute to understanding how cadmium exposure affects molecular responses in bioindicator species, and evaluate the feasibility of using *C. fluminea* for monitoring cadmium pollution, particularly in anthropogenically impacted rivers like the Euphrates.

## Methods

**Study design:** this study utilised a cross-sectional design to assess spatial variation in cadmium exposure and its impact on antioxidant gene expression in *Corbicula fluminea*. A cross-sectional approach was chosen to capture a snapshot of cadmium concentrations and biological responses across three distinct sites along the Euphrates River at a single point in time. This design allowed for the examination of natural cadmium exposure in areas with varying levels of anthropogenic influences.

**Study setting:** the study was conducted along a 26-kilometer stretch of the Euphrates River in southern Iraq. Three study sites were selected based on differing anthropogenic activities, which could influence cadmium contamination. The sites were:

***Nasiriya centre:*** an urbanised area heavily impacted by industrial discharge and domestic runoff.

***Fadliah:*** an agricultural area affected by runoff from fertilisers and pesticides, which may influence metal concentrations.

***Suq Al-Shuyoukh:*** a region dominated by aquaculture activities, particularly fish breeding, influencing water quality through organic waste and effluents.

These sites were chosen to represent different levels of environmental exposure, providing a broad perspective on how cadmium concentrations and biological responses vary with anthropogenic activity in the region.

**Participants:** the study focused on *Corbicula fluminea* specimens collected from the three study sites. Only adult clams with shell lengths between 2 and 5 cm were included to ensure consistency in size and maturity. Clams that showed signs of disease or physical damage were excluded to minimise confounding factors that could affect the molecular analysis. A total of 90 clams (30 from each site) were collected using the standardised quadrat sampling method, ensuring consistent sampling across sites. The clams were transported to the laboratory under chilled conditions to preserve tissue integrity.

**Interventions and comparisons:** the study aimed to observe the natural exposure of *C. fluminea* to cadmium across the three sites. No controlled interventions were applied to the clams, and the focus was on examining natural cadmium exposure. Comparisons were made between sites based on cadmium concentrations in both water and clam tissues, as well as gene expression levels of antioxidant genes, including Metallothionein (MT), Glutathione-S-transferase (GST), Catalase (CAT), and Superoxide Dismutase (SOD).

### Data collection

**Cadmium quantification:** water samples (1 liter per site) were collected approximately 15 cm below the surface to avoid surface debris. The samples were acidified with nitric acid (5 mL of 55% nitric acid per liter) to prevent metal adsorption and were stored at 4°C until analysis. Tissue samples were dissected from clams, freeze-dried, and homogenised for cadmium quantification. Both water and tissue samples were analysed for cadmium concentrations using Atomic Absorption Spectroscopy (AAS). Cadmium analysis was performed using a flame Atomic Absorption Spectrophotometer (model and manufacturer), following standard procedures described in APHA guidelines (APHA, 2017).

**Gene expression analysis:** to analyse gene expression, RNA was extracted from 100 mg of clam tissue using TRI Reagent® and a bead mill homogenizer. The purity and integrity of the RNA were assessed using spectrophotometry and agarose gel electrophoresis. cDNA synthesis was performed using the Prime Script^TM^ RT reagent kit, following the manufacturer's protocols. Quantitative PCR (qPCR) was carried out using KAPA SYBR® FAST Master Mix and gene-specific primers for MT, GST, CAT, and SOD, with ACT serving as the reference gene. qPCR amplification conditions followed manufacturer recommendations and previously validated protocols for antioxidant gene expression analysis in bivalves [8]. The relative gene expression levels were calculated using the CT method. This method compares the cycle threshold (Ct) values of the target gene and the reference gene to quantify the relative gene expression between samples, normalising the data to control for any variations in cDNA amounts.

**Environmental parameters:** during sampling, key environmental parameters such as temperature, pH, and salinity were measured using a multiparameter water quality meter. These parameters were documented to identify potential environmental confounders that might influence cadmium exposure or gene expression.

**Statistical methods:** data analysis was performed using SPSS version 26. Descriptive statistics, including means and standard deviations, were calculated for cadmium concentrations and gene expression levels. One-way ANOVA was used to compare cadmium concentrations and gene expression across the three study sites, with post-hoc pairwise comparisons conducted using Tukey's Honestly Significant Difference (HSD) test. Pearson correlation was applied to assess the relationship between cadmium concentrations (in both water and tissues) and the expression of antioxidant genes. A power analysis was conducted using G*Power 3.1 to estimate the required sample size, which was based on a medium effect size (f = 0.25) with an alpha level of 0.05 and a power of 80%. It was determined that 30 clams per site would be sufficient to detect statistically significant differences. The significance level for all tests was set at p < 0.05, and all data were checked for normality and homogeneity of variances before analysis.

## Results

**Figure 1:**
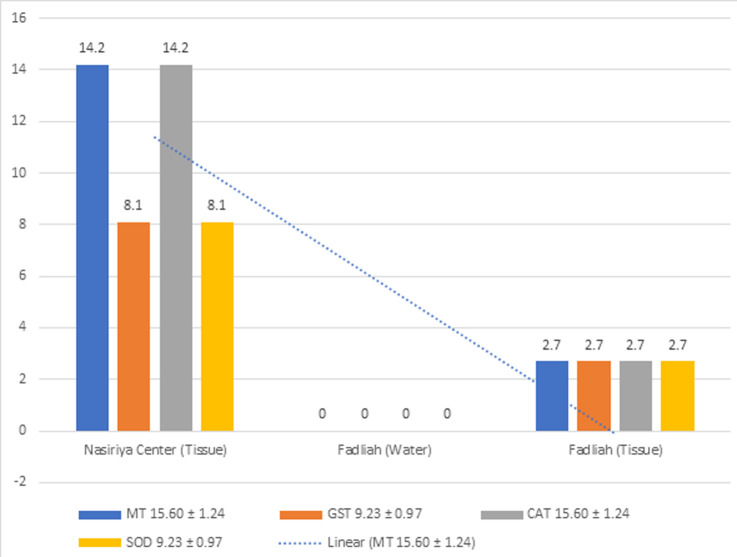
relative gene expression levels (∆Ct values ± SD) of MT, GST, CAT, and SOD in *C. fluminea* collected from three study sites along the Euphrates River

**Participants and sample exclusion:** a total of 90 *Corbicula fluminea* specimens were initially collected from three sites along the Euphrates River: Nasiriya Centre (urban), Faliha (agricultural), and Siq Al-Shayok (aquaculture). Thirty clams were sampled from each site. However, due to RNA degradation, physical damage, and sample loss during transportation, only 9 clams (3 from each site) met RNA quality control criteria and were included in the final gene expression analysis. Therefore, the final sample consisted of 9 clams, one from each site, for gene expression analysis. The sampling process is visually summarised in Figure 1.

**Exclusion criteria:** of the 90 clams collected, 17 were excluded due to insufficient RNA quality, and 3 were excluded because of physical damage. Consequently, only 10% of collected samples passed RNA quality control and were included in gene expression analysis, which was a result of strict quality control measures to ensure accurate molecular data.

**Environmental conditions and cadmium exposure:** the environmental conditions at the three study sites varied considerably in terms of cadmium exposure and key parameters such as temperature, pH, and salinity. Table 1 presents these environmental conditions: Nasiriya Centre, an urbanised area, had the highest cadmium concentrations, followed by Siq Al-Shuyoukh, an aquaculture site, and Fadliah, an agricultural site. Figure 1 and Table 2 show the cadmium concentrations in both water and tissues from each site. Cadmium levels were significantly higher at Nasiriya Centre in both water (mean: 15.60 µg/g) and tissue samples (mean: 14.2 µg/g), likely due to industrial discharge and domestic runoff. Faliha had the lowest cadmium concentrations in both water (mean: 2.60 µg/g) and tissue (mean: 2.7 µg/g), reflecting the agricultural setting with contamination primarily from fertiliser and pesticide runoff. Siq Al-Shayok had intermediate cadmium concentrations in both water (mean: 9.23 µg/g) and tissue (mean: 8.1 µg/g), possibly influenced by waste from fish farming.

**Table 1:** environmental conditions at three study sites along the Euphrates River (sampling dates: Nasiriya Center, 05-03-25; Fadliah, 07-03-25; Suq Al-Shuyoukh, 09-03-25)

Site	Temperature (°C)	pH	Salinity (ppt)
Nasiriya Center	27.2 ± 2.4	7.6 ± 0.1	2.9 ± 0.5
Fadliah	25.8 ± 1.9	7.4 ± 0.3	1.5 ± 0.3
Suq Al-Shuyoukh	28.1 ± 2.1	7.8 ± 0.2	3.2 ± 0.4

**Table 2:** cadmium concentrations in water (µg/L) and clam tissue (µg/g dry weight) at three study sites along the Euphrates River (sampling date: 05-03-25)

Site	Water (µg/L)	Tissue (µg/g dry weight)
Nasiriya Center	15.60 ± 1.24	14.2
Fadliah	2.60 ± 0.43	2.7
Suq Al-Shuyoukh	9.23 ± 0.97	8.1

**Gene expression analysis:** gene expression levels for four key antioxidant genes-Metallothionein (MT), Glutathione-S-transferase (GST), Catalase (CAT), and Superoxide Dismutase (SOD)-were measured using quantitative PCR (qPCR). Despite differences in cadmium exposure across sites, no significant variations in gene expression were observed between the three sites. MT expression showed no significant differences between the sites, with slightly higher expression at Nasiriya Centre (0.066 ± 0.18) compared to Faliha (0.10 ± 0.15) and Siq Al-Shayok (0.0001 ± 0.36). The p-value (p = 0.9600) indicated no statistical significance. GST expression was highest at Siq Al-Shayok (0.23 ± 0.37), but no significant differences were observed across the sites (p = 0.3910). CAT expression levels were similar at all sites, with no significant differences found (p = 0.7350), suggesting that cadmium exposure did not induce a noticeable increase in CAT expression. SOD expression was also similar across the three sites, with levels of -0.03 ± 0.26 at Nasiriya Centre, 0.16 ± 0.33 at Faliha, and 0.03 ± 0.13 at Siq Al-Shayok. The p-value (p = 0.8950) confirmed no significant differences. These results suggest that despite marked differences in cadmium exposure, *C. fluminea* did not show significant gene expression changes for the antioxidant genes measured, indicating a limited molecular response to cadmium contamination in natural conditions.

**Correlation between cadmium levels and gene expression:** Pearson correlation analyses were performed to examine the relationship between cadmium concentrations (in both water and tissues) and the expression of the antioxidant genes. No significant correlations were found, as summarised below: MT: r = 0.085, p = 0.828; GST: r = 0.182, p = 0.639; CAT: r = -0.018, p = 0.963; SOD: r = -0.008, p = 0.984. These results indicate that cadmium exposure, whether measured in water or tissue samples, did not directly affect the expression of the selected antioxidant genes. This suggests that the molecular response to cadmium exposure might be influenced by factors other than the cadmium concentrations themselves, such as adaptive mechanisms or interactions with other pollutants.

**Influence of environmental parameters on gene expression:** further analyses explored whether environmental factors such as pH, temperature, and salinity influenced gene expression. However, no significant correlations were found between these parameters and the expression of antioxidant genes: pH: r = -0.052, p = 0.926; Temperature: r = 0.103, p = 0.712 and Salinity: r = -0.129, p = 0.645. These results suggest that environmental conditions (pH, temperature, and salinity) did not have a significant impact on gene expression in *C. fluminea* under the conditions of this study.

**Summary of findings:** Cadmium concentrations showed significant spatial variation across the three sites, with the highest concentrations observed at Nasiriya Centre and the lowest at Faliha. Gene expression for MT, GST, CAT, and SOD did not show significant upregulation across sites, despite the observed variation in cadmium exposure. Pearson correlation analyses demonstrated no significant relationship between cadmium levels and gene expression, suggesting that other factors might be influencing gene expression responses. Environmental parameters such as pH, temperature, and salinity did not significantly correlate with gene expression, further suggesting that additional factors may contribute to the observed molecular responses.

Despite significant spatial variation in cadmium contamination across the three study sites, *C. fluminea* did not exhibit a strong molecular response at the gene expression level. This suggests that the antioxidant genes measured may not be reliable biomarkers for cadmium exposure under natural environmental conditions. The absence of significant upregulation of these genes, despite varying cadmium levels, could indicate the presence of adaptive mechanisms that mitigate cadmium toxicity, which are not reflected at the gene expression level.

Further research is needed to explore alternative biomarkers or molecular responses, such as protein activity or enzymatic assays, that may provide a clearer understanding of cadmium exposure in aquatic organisms. Additionally, considering multiple pollutant exposures and the long-term effects of cadmium on bioindicator species in dynamic environments like the Euphrates River will be crucial for a more comprehensive assessment of environmental health.

## Discussion

**Key findings:** this study aimed to examine spatial variation in cadmium exposure and its impact on antioxidant gene expression in *Corbicula fluminea* across three sites along the Euphrates River. The main findings are:

***Spatial variation in cadmium exposure:*** cadmium concentrations were significantly higher in Nasiriya Centre (urban site) compared to Faliha (agricultural site) and Siq Al-Shayok (aquaculture site). Nasiriya Centre exhibited the highest concentrations in both water and clam tissues, followed by Siq Al-Shayok and Fadliah, which had the lowest concentrations.

***Gene expression results:*** despite differences in cadmium levels, there were no significant changes in the expression of MT, GST, CAT, or SOD across the sites. This indicates that the antioxidant gene responses were not significantly influenced by cadmium exposure in natural conditions.

***Lack of correlation:*** Pearson correlation analysis revealed no significant relationships between cadmium concentrations and the expression of antioxidant genes. This suggests that other factors, beyond cadmium exposure, may affect the molecular responses of *C. fluminea*.

**Comparison with existing literature:** our findings are consistent with some studies but also diverge from others [9]. Laboratory studies, such as those observed upregulation of antioxidant genes like MT, GST, SOD, and CAT in clams and fish exposed to cadmium in controlled environments [10]. However, in this field-based study, *C. fluminea* did not exhibit significant molecular changes despite similar cadmium exposure gradients [11]. This discrepancy may be due to the complexity of natural environments, where multiple stressors affect biological responses, unlike controlled laboratory conditions. Furthermore, our findings align with Papaioannou *et al*. [12], who reported limited correlation between heavy metal concentrations and oxidative stress biomarkers in field-collected bivalves. This highlights the challenge of interpreting molecular responses in real-world conditions, where environmental factors like temperature, salinity, and species-specific adaptations may play a role [13].

**Significance of results:** the absence of significant gene expression changes, despite the observed cadmium contamination, suggests that *C. fluminea* may exhibit adaptive mechanisms not detectable at the gene expression level. For example, the species could employ metal sequestration, enhanced detoxification pathways, or cellular repair mechanisms to mitigate cadmium's toxic effects [14]. The absence of significant gene expression differences may reflect adaptive tolerance mechanisms developed through chronic low-level cadmium exposure. Long-term exposure can lead to physiological acclimation, reducing the need for acute transcriptional upregulation of antioxidant genes. Additionally, interactions with other environmental stressors may modulate gene regulation pathways, potentially masking cadmium-specific effects and complicating the interpretation of biomarker responses in natural ecosystems [15].

This finding is important because it suggests that antioxidant gene expression alone may not be a reliable biomarker for cadmium exposure under field conditions, particularly in ecosystems influenced by multiple environmental stressors [16]. In controlled laboratory settings, gene expression changes are commonly used to assess pollutant exposure; however, the results of this study indicate that real-world conditions are considerably more complex [17]. *C. fluminea* may have developed tolerance strategies that are not directly reflected in transcriptional activity. Future studies should therefore consider additional biomarkers, such as protein expression levels, enzyme activity assays, metallothionein quantification, or oxidative stress indicators, to provide a more comprehensive understanding of the biological effects of pollutants [18].

**Strengths and limitations:** this study's key strength is its field-based design, providing real-world insights into the biological responses of *C. fluminea* to cadmium exposure [19]. By examining sites with varying levels of anthropogenic influence, the study offers valuable information on the potential use of *C. fluminea* as a bioindicator species for cadmium contamination.

However, several limitations should be acknowledged:

***Small sample size:*** the limited number of samples (n = 9) substantially reduced statistical power and increased the risk of Type II error, potentially masking subtle but biologically relevant gene expression differences.

***Cross-sectional design:*** the cross-sectional nature of the study prevents causal inferences about the relationship between cadmium exposure and gene expression. Longitudinal studies would help capture temporal effects and provide a clearer understanding of the long-term impacts of cadmium on gene expression.

***Environmental confounders:*** while environmental parameters like pH, temperature, and salinity were measured, their potential effects on gene expression were minimal. Other unmeasured factors, such as nutrient pollution or microplastic contamination, could have influenced the results.

***Absence of protein-level data:*** gene expression alone may not fully capture the biological response to cadmium exposure. Future studies should include protein-level analysis or enzyme activity assays to offer a more complete picture of detoxification and oxidative stress responses.

From an ecosystem perspective, elevated cadmium levels observed at urban and aquaculture sites may pose risks not only to aquatic biodiversity but also to human populations relying on the Euphrates River for domestic and agricultural purposes. Bioaccumulation of cadmium in edible aquatic species could contribute to chronic dietary exposure. Therefore, continuous monitoring of heavy metals in freshwater systems remains essential for environmental protection and public health management [20].

**Future research directions:** based on the findings of this study, future research should focus on:

***Longitudinal studies:*** to assess the long-term effects of cadmium exposure on gene expression and other biomarkers in *C. fluminea*.

***Multiple pollutant exposure:*** investigating the combined effects of cadmium and other environmental contaminants (e.g., heavy metals, pesticides, microplastics) will provide a more comprehensive understanding of pollutant interactions and their cumulative impacts on biological systems.

***Molecular-level investigations:*** future studies should incorporate protein activity assays and enzyme function analysis to better understand detoxification mechanisms in *C. fluminea*.

***Species-specific variations:*** further research should explore inter-species variability in antioxidant gene expression to determine how generalizable *C. fluminea* is a bioindicator across different ecosystems.

**Generalisability:** while this study provides valuable insights into the biological responses of *C. fluminea* to cadmium exposure in the Euphrates River, the results may not be universally applicable [21]. The findings should be interpreted within the context of this specific river system, with consideration for species-specific responses and local pollution profiles [22]. Comparative studies in other rivers and ecosystems will help validate the potential of *C. fluminea* as a bioindicator for cadmium contamination. Despite significant spatial variation in cadmium contamination across the study sites, *C. fluminea* did not exhibit a strong molecular response at the gene expression level [23]. This suggests the presence of adaptive mechanisms that mitigate cadmium toxicity, which are not detectable through gene expression alone [24]. Future research should explore alternative biomarkers, such as protein activity assays, and investigate the combined effects of multiple pollutants to provide a more comprehensive understanding of the impacts of cadmium exposure in aquatic ecosystems.

## Conclusion

This study investigated the spatial variation in cadmium exposure and its impact on antioxidant gene expression in *Corbicula fluminea* from three sites along the Euphrates River. The key findings include significant spatial differences in cadmium concentrations, with Nasiriya Centre exhibiting the highest levels, followed by Siq Al-Shayok and Faliha. However, despite these differences in exposure, no significant changes in the expression of MT, GST, CAT, or SOD genes were observed across sites, suggesting that *C. fluminea* may not exhibit a robust molecular response to cadmium under the natural environmental conditions studied. The lack of significant gene expression changes, despite varying cadmium levels, raises questions about the sensitivity of *C. fluminea* as a bioindicator species for cadmium pollution in field settings, particularly when multiple stressors may influence biological responses. The findings imply that antioxidant gene expression alone may not be sufficient for assessing pollutant impacts in complex environmental contexts. Given these results, future research should focus on longitudinal studies to evaluate the temporal effects of cadmium exposure and incorporate additional biomarkers such as protein activity and enzyme function to provide a more comprehensive understanding of biological responses. Furthermore, multi-pollutant exposure studies are needed to examine the combined effects of cadmium and other contaminants that may influence gene expression in bioindicator species. From a policy perspective, the study highlights the need for enhanced monitoring of heavy metal pollution in freshwater systems, particularly in urbanised and agriculturally impacted regions. The integration of bioindicator species like *C. fluminea* into environmental monitoring programs can provide valuable insights into the health of aquatic ecosystems and inform strategies for mitigating pollution. However, a broader set of biomarkers and environmental variables should be considered to better understand the complex interactions between pollutants and biological systems. The findings reinforce the importance of integrating molecular biomarkers with physicochemical monitoring to strengthen environmental surveillance frameworks in freshwater ecosystems affected by anthropogenic activities.

### What is known about this topic

- Cadmium is a toxic heavy metal that accumulates in aquatic ecosystems, primarily through industrial effluents, agricultural runoff, and waste discharge, leading to oxidative stress in aquatic organisms;
- Corbicula fluminea is a well-established bioindicator species used to monitor heavy metal contamination in freshwater systems due to its ability to accumulate pollutants from the water;
- Oxidative stress biomarkers, including metallothionein (MT), glutathione-S-transferase (GST), superoxide dismutase (SOD), and catalase (CAT), are commonly used to assess the biological impacts of cadmium exposure in aquatic organisms.

### What this study adds

- This study provides field-based evidence on the spatial variation in cadmium exposure along the Euphrates River and its impact on antioxidant gene expression in C. fluminea, highlighting the complexity of pollutant effects in natural environments;
- The study demonstrates that gene expression changes in C. fluminea do not always correlate with cadmium concentrations, suggesting that other environmental factors or adaptive responses may influence biomarker expression;
- This research contributes to the evaluation of bioindicator species for cadmium monitoring, suggesting the need for multiple biomarker approaches to assess the effects of pollution in complex aquatic ecosystems.
